# RNA-aptamers-in-droplets (RAPID) high-throughput screening for secretory phenotypes

**DOI:** 10.1038/s41467-017-00425-7

**Published:** 2017-08-23

**Authors:** Joseph Abatemarco, Maen F. Sarhan, James M. Wagner, Jyun-Liang Lin, Leqian Liu, Wafa Hassouneh, Shuo-Fu Yuan, Hal S. Alper, Adam R. Abate

**Affiliations:** 1 Department of Chemical Engineering, The University of Texas at Austin, 200 E Dean Keeton St Stop C0400, Austin, Texas 78712 USA; 2 Bioengineering and Therapeutic Sciences, University of California San Francisco, San Francisco, 94158 California USA; 3 California Institute for Quantitative Biosciences, University of California San Francisco, San Francisco, 94158 California USA; 4 Chan Zuckerberg Biohub, San Francisco, 94158 California USA; 5 Institute for Cellular and Molecular Biology, The University of Texas at Austin, 2500 Speedway Avenue, Austin, Texas 78712 USA

## Abstract

Synthetic biology and metabolic engineering seek to re-engineer microbes into “living foundries” for the production of high value chemicals. Through a “design-build-test” cycle paradigm, massive libraries of genetically engineered microbes can be constructed and tested for metabolite overproduction and secretion. However, library generation capacity outpaces the rate of high-throughput testing and screening. Well plate assays are flexible but with limited throughput, whereas droplet microfluidic techniques are ultrahigh-throughput but require a custom assay for each target. Here we present RNA-aptamers-in-droplets (RAPID), a method that greatly expands the generality of ultrahigh-throughput microfluidic screening. Using aptamers, we transduce extracellular product titer into fluorescence, allowing ultrahigh-throughput screening of millions of variants. We demonstrate the RAPID approach by enhancing production of tyrosine and secretion of a recombinant protein in *Saccharomyces cerevisiae* by up to 28- and 3-fold, respectively. Aptamers-in-droplets affords a general approach for evolving microbes to synthesize and secrete value-added chemicals.

## Abstract (web summary)

Screening libraries of genetically engineered microbes for secreted products is limited by the available assay throughput. Here the authors combine aptamer-based fluorescent detection with droplet microfluidics to achieve high throughput screening of yeast strains engineered for enhanced tyrosine or streptavidin production.

## Introduction

Microbes can perform chemical transformations with an ease and elegance that often outclasses the best synthetic chemistry techniques. They operate on a chemical palette that dwarfs the diversity that is available from petrochemicals, and they accomplish these feats in an aqueous environment at room temperature and pressure. A major motivation of synthetic biology is, thus, to re-engineer natural microbes into “living foundries” that produce high value chemicals from renewable resources. Synthetic biology embraces a design-build-test cycle to engineer such non-native phenotypes into microbes, for the betterment of humankind and the environment. By utilizing metabolic engineering principles, it is possible to address grand challenges, including establishing sustainable alternatives to a fossil-fuel reliant chemical industry^1, 2^. In recent years, the capacity to design and build strains has accelerated with advances in DNA assembly and synthesis^3, 4^, genome editing^5–8^, in vivo evolution^9, 10^, computational design of synthetic circuits^11–13^, synthetic regulatory and perturbation systems^14–17^, and automation^18–20^. Despite these advances, the phenotypic test step in which each variant is assayed is a common bottleneck, often lagging by orders-of-magnitude the throughput with which we can design and build libraries. Consequently, there has been major investment in the development of technologies for rapid testing of library variants. However, traditional chromatography-reliant metabolite detection modalities such as gas chromatography-mass spectrometry (GC-MS) and high pressure liquid chromatography–mass spectrometry (HPLC-MS) are too slow without brute-force parallelization and associated high costs. Alternative approaches, such as protein-based biosensors, can link target metabolite concentration to fluorescence or growth-selectable traits and thus enable more rapid and efficient screening^21–24^. However, biosensor development can be laborious for each new target and often requires host-cell modifications or DNA rewiring. Thus, new approaches for more general target detection are required to speed the test portion of the prototyping cycle.

A complicating challenge is that many metabolites of interest are secreted and, thus, the production phenotype is not directly evolvable by growth selection or flow cytometric screening. To identify a high extracellular producer, the secreted product from that producer must be physically isolated from that of all others, so it can be associated to the corresponding cell. One approach is to perform single strain assays in microtiter plates, but this is generally limited to hundreds to thousands of strains. Droplet microfluidic platforms shatter this barrier, as they are capable of rapidly culturing, isolating^25–28^, and screening millions of cells per hour based on secreted product titers^29–33^ at a fraction of the cost of well plate methods^34^. Despite these advantages, however, a critical limitation remains: product concentration must be measurable via fluorescence, the only current modality with sufficient signal to robustly detect in kilohertz flowing droplets. The vast majority of target molecules, however, are non-fluorescent and non-trivial to couple to a fluorescence assay, limiting droplet screening to proof-of-principle niche applications. To enable more effective engineering of microbes, general and high-throughput evolution and screening strategies are needed.

**Fig. 1:**
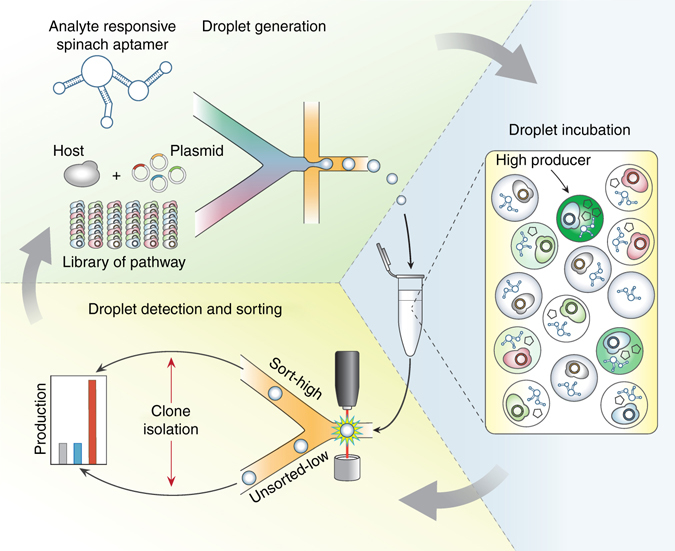
Overview of RAPID Screening. RNA-aptamers-in-droplets (RAPID) screening uses analyte-responsive RNA aptamers grafted to the Spinach aptamer backbone to detect analyte concentrations in microdroplets. The aptamer is co-encapsulated with a member of a yeast mutant library and incubated to produce the molecule of interest and develop a fluorescence signal. Droplets then flow through a microfluidic device and are sorted based on fluorescence using dielectrophoresis. Improved variants are recovered and the evolution cycle can be repeated if desired

In this paper, we present RNA-aptamers-in-droplets (RAPID, Fig. 1), a general method for harnessing the power of ultrahigh-throughput droplet screening for the enhancement of secretory phenotypes. The core innovation of RAPID is the use of “Spinach” aptamers^35^ to transduce secreted target molecule concentration into a fluorescence signal appropriate for kilohertz droplet sorting. Aptamers are amazingly general sensors, with hundreds of sequences reported in the literature with characterized binding affinity and kinetics toward analytes of biotechnological interest, including small molecules and proteins^36^. Quantitative dose-response relationships have also been established for many of these aptamers^35, 37^. They thus provide a general sensing technology for customizing RAPID screening of diverse molecular targets, by changing only the aptamer sequence. We use RAPID screening to engineer *Saccharomyces cerevisiae* strains with enhanced extracellular production of a small molecule metabolite (tyrosine) and enhanced secretion of a recombinant protein (streptavidin). This work establishes RAPID as a general, high-throughput screening method for microbial strain development, protein engineering, and synthetic biology.

## Results

### Design criteria for RAPID screening

RAPID is a general approach for enhancing the production of non-fluorescent target molecules with droplet microfluidic screening. To apply RAPID, several conditions must be met: (i) single cells must be cultivated in a metabolically active and productive state in picoliter droplets; (ii) RNA aptamers must allow target concentration measurements in picoliter droplets; (iii) the aptamer must remain sensitive and specific even in the presence of the producer cell and growth medium for the several day production phase; and (iv) the microfluidic system must perform all requisite operations efficiently and allow recovery of live cells for downstream cultivation and sequencing analysis of responsible proteins or pathways.

### Yeast cells proliferate and produce in microfluidic droplets

Differentiating between cells that produce large quantities of target molecule and those that produce little requires incubating the cells to allow high producers to fill their droplets to a detectable product concentration. Hence, a first condition for RAPID is that cells must live and produce in the droplets for the required multi-day incubation. To test the viability and productivity of *Saccharomyces cerevisiae* in picoliter droplets, we cultivated a strain producing the yellow fluorescent protein yECitrine. The cells proliferated readily and fluoresced brightly (Supplementary Fig. 1), thus demonstrating their metabolically active state. This shows that the droplet environment is suitable for multi-day yeast culture and production which will enable droplets to be sorted based on fluorescence, validating that our specific microfluidic platform matches previously reported capabilities of microfluidic droplets^29–33^.

### RNA aptamers sense a variety of target molecules in droplets

**Fig. 2:**
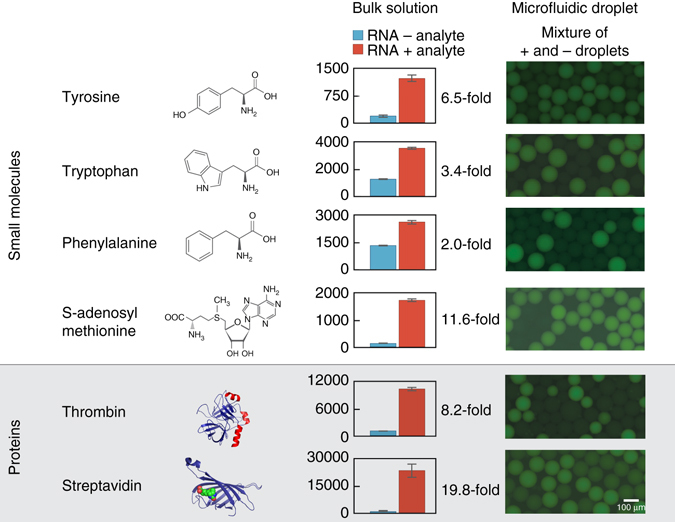
Establishing a panel of Spinach-based aptamers that can detect analytes in droplets. A panel of Spinach-based aptamer sensors was tested across a variety of small molecule and protein targets. Aptamers were synthesized through in vitro transcription and incubated with or without the analyte of interest, and fluorescence was measured. *Bar graphs* represent the mean of three replicates (*error bars* depict s.d.). On the right, solutions of dye with RNA aptamer or with both RNA aptamer and analyte were encapsulated and the resulting microdroplets were imaged using fluorescence microscopy. The negative (RNA alone) and positive (RNA and analyte) droplets were mixed together for simultaneous imaging of contrast. All images were acquired under the same magnification, and representative images from multiple independent trial runs are depicted. Sequences of all aptamers used in this study can be found in Supplementary Table 1

The key innovation of RAPID that makes it general is the use of Spinach-based aptamers to transduce extracellular metabolite concentration into an optically measurable fluorescence signal^35^. Spinach aptamers are a modular sensing technology consisting of an RNA molecule that can bind an exogenous dye and target ligand to yield a fluorescence signal. The RNA sequence can be reprogrammed to recognize diverse targets, including amino acids, nucleotides, and even proteins (Supplementary Fig. 2)^35, 37^. Sensing aptamers can also be constructed of DNA using conjugation with fluorophore and quencher combinations^38^, but this approach typically requires covalent modification of the aptamer. In contrast, Spinach RNA aptamers require only a short DNA template (100–200 bp), a commercial in vitro transcription kit, and a commercially-available universal dye; thus, RNA aptamers provide a general and facile assay for use in droplets. To illustrate this concept, we created a panel of aptamers targeting an array of small molecules and proteins (Fig. 2). In all cases, the reported analyte-binding aptamer was grafted to the modular Spinach or Spinach2 domain (Supplementary Fig. 2, Supplementary Table 1). Several designs had never been attempted experimentally, and thus we performed thermodynamic optimization using mfold^39^, arriving at several candidates for testing. We then tested the best performers from the computational screen in droplet assays. In our first droplet tests, however, we found that aptamers performing well in bulk performed poorly in droplets, particularly when incubated with cells. We thus optimized assay conditions for the tyrosine aptamer until we identified ones that yielded sensitive and stable tyrosine detection in droplets (Supplementary Fig. 3). Under these fixed conditions, aptamers afford an amazingly general sensing modality for microfluidic droplets (Fig. 2), providing a “plug-and-play” approach for sensing diverse target molecules by swapping only aptamer sequence.

### Aptamers sense molecules secreted by cells in droplets

During screening, the yeast cells are cultured in droplets in a productive metabolic state to secrete the target molecule. The secreted target molecule, in turn, interacts with the Spinach aptamer in solution, generating a fluorescence signal. Hence, the detection aptamer should ideally be stable in the droplet over the several day production phase and in the presence of cells. Otherwise, the aptamer would need to be added after production using more advanced and less readily available microfluidic approaches such as droplet merging or picoinjection^40, 41^. Reliance on more complex microfluidic devices could limit the applicability and adoption of RAPID, so we sought to achieve stable aptamer performance in the presence of cells.

Initially, incubations of analyte and cells significantly hampered aptamer signal development. To address this, we tested blocking agents and found that 0.1 mg ml^−1^ of double-stranded salmon sperm DNA minimized interactions with the cell and maintained stable aptamer signals (Supplementary Fig. 4). To identify tyrosine-responsive aptamer variants capable of surviving incubation with cells in droplets, we used a Spinach-tyrosine sensor consisting of tyrosine aptamer Tyr1^42^, the Spinach2 stem loop backbone, and variants of a signal transducing stem. Similar to previous Spinach biosensor development efforts that tested 7–10 variants^35^, we tested seven variants to identify one (Tyr1M1) that was sufficiently sensitive, stable, and inducible (Supplementary Fig. 5). We encapsulated this Tyr1M1 aptamer in droplets with a panel of engineered yeast strains secreting varying levels of tyrosine^43, 44^ and found that, indeed, the Tyr1M1 aptamer allowed reliable identification of strains based on their extracellular tyrosine production. We confirmed these results by comparing the aptamer assay with an independent chemical derivatization assay performed on the different strains, and found good correlation between the assays (Pearson’s *r* = 0.9175, *p* = 0.0036; Supplementary Fig. 6). This demonstrates that Tyr1M1 is effective for differentiating between yeast cells based on tyrosine production and, thus, can be used for ultrahigh-throughput single-cell screening to identify high producers in a randomized library.

### Enhancing tyrosine production with RAPID evolution

Tyrosine and other shikimate-derived molecules have multiple uses as polymer precursors, nutritional supplements, and therapeutic agents, but suffer from low yields in yeast due to tight regulatory control^45^. We sought to use RAPID screening to evolve a well-characterized, feedback sensitive rate-limiting enzyme in the pathway, the 3-deoxy-D-arabino-heptulosonate-7-phosphate (DAHP) synthase encoded by the gene *aro4*. Prior work demonstrated that several point mutations in this key enzyme, especially K229L^46, 47^, reduce feedback inhibition and improve tyrosine production^47^. To both de novo identify new mutations and further improve upon the best-reported Aro4p K229L mutant, we established two directed evolution libraries that targeted: (1) specifically the regulatory region of the wild-type protein (residues 191–263) and (2) the entire protein sequence in the background of the K229L mutant. While this initial example targeted *aro4* for directed evolution, the RAPID approach can also be applied to screen diversity in other genes, pathways, or even the whole genome.

**Fig. 3:**
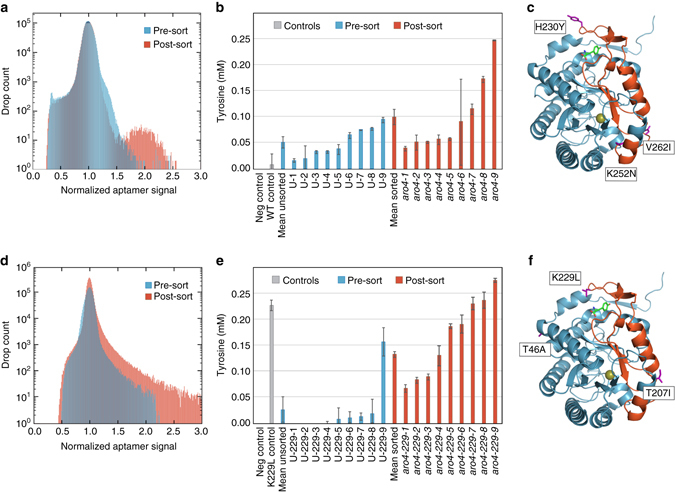
RAPID screening for the improvement of tyrosine production through an evolved *Aro4p*. The RAPID screening approach was used to identify novel mutations in *aro4* that improve tyrosine production by yeast. Libraries were constructed through mutagenizing the proposed regulatory region of Aro4p (residues 191–263) **a**–**c** and through whole-gene mutagenesis of the best previously reported variant *aro4-K229L* **d**–**f**. Histograms of droplet fluorescence pre-sort and post-sort (re-encapsulated) demonstrate enrichment through the process (**a**, **d**). Isolated and re-transformed clones randomly selected from the pre- and post-sort populations were quantified for tyrosine production with the sorted clones having a 1.9- and 5.8-fold increase in secretion compared to the unsorted clones for Aro4p and K229L clones, respectively (**b**, **e**). Crystal structures of Aro4p are derived from PDB 1OF6 and marked with the identified mutations (**c**, **f**). *Error bars* represent standard error of biological triplicates. Tyrosine production was increased nearly 28-fold over the wild-type production using this approach

Our libraries consisted of ~ 10^5^ mutants, which we encapsulated in droplets with the Spinach-Tyr1M1 aptamer and dye, and incubated for tyrosine production. The brightest 0.3–0.5% of droplets were recovered via fluorescence-activated droplet sorting, and the obtained mutant sequences were analyzed. Droplet fluorescence distributions indicate significant enrichment for improved tyrosine production post-sorting (Fig. 3a, d; Supplementary Fig. 7). To confirm this, we re-transformed the sorted and unsorted variants into a fresh yeast strain to eliminate possible strain adaptations that may have occurred during the process, and assayed the re-transformed clones for extracellular tyrosine production^45^. The sorted variants were statistically enriched for improved production over randomly selected variants from the library (*p* < 0.05, Mann–Whitney *U*–test; Figs. 3b, e). Additionally, the mean secreted tyrosine titer of sorted clones was two (Fig. 3b) to fivefold (Fig. 3e) improved relative to the mean titer of clones picked randomly from the unsorted library population. The top performing variant from each library contained novel mutations and was improved over the best known *aro4-K229L* mutant. The mutant *aro4-9* derived from wild-type *ARO4* (library 1) was 27-fold improved over wild-type and has three coding mutations (H230Y, K252N, and V262I; Fig. 3c). We investigated this variant further and determined that the improvement is primarily driven by the novel H230Y mutation, a residue adjacent to the reported K229 mutation (Supplementary Fig. 8). The mutant *aro4-229-9* derived from further mutagenesis of *aro4-K229L* (library 2) showed a 28-fold improvement over wild type and a 21% improvement relative to *aro4-K229L*, and has two additional coding mutations (T46A and T207I, Fig. 3f).

Importantly, while each improved enzyme possesses novel mutations that increase tyrosine production, cell growth rate is not affected and, thus, these variants could not have been enriched for and discovered via growth selections (Supplementary Fig. 9). Furthermore, because it has not been previously possible to screen for the impact of *aro4* in a secretion pathway in a high-throughput format, all identified mutations were previously unknown. Indeed, previous efforts to enhance *aro4* relied on hypotheses based on crystal structures and understandings of protein dynamics^46^, approaches that are not always possible or do not always yield the optimal structure, particularly if the enzyme to be evolved is difficult to crystallize, poorly understood, or embedded in a multi-component pathway. The RAPID approach is thus a powerful alternative because it allows discovery of unknown and unpredictable mutations in an unbiased manner.

### Enhancing streptavidin secretion with RAPID screening

To illustrate the generality of the RAPID screening approach and aptamer sensing, we used RAPID to enhance a different phenotype; the secretion of a recombinant protein via secretory tag directed evolution. Despite the markedly different biological objective of this screen, the RAPID process is virtually unchanged except in the aptamer ligand-binding domain, further illustrating the flexibility of the method. As a model recombinant protein target, we used streptavidin, a well-characterized protein derived from *Streptomyces avidinii* that binds with high affinity and specificity to biotin and peptide affinity tags^48, 49^ and has wide-ranging biotechnological applications^50–54^.

Previous attempts for optimizing heterologous expression of full-length streptavidin have been conducted in bacteria such as *E. coli* or *B. subtilis* ^55, 56^ and in the yeasts *Saccharomyces cerevisiae* or *Pichia pastoris* ^57, 58^ achieving titers of up to 62 mg l^−1^ in *P. pastoris* after clone screening and optimization^57^. To secrete protein in *S. cerevisiae*, a secretion tag is required and, thus, we use RAPID to evolve the commonly used *α*-mating factor secretory leader (αMF). Mutants of this sequence have been investigated for improving secretion of antibody fragments (scFv)^59^, but the screening process required a complex chemical conjugation to alter the cell surface and was dependent on antibody binding. By contrast, RAPID screening requires no chemical modification of production cells and is not dependent on properties of the secreted recombinant protein. These advantages allow evolutionary selection that better co-evolves secretion signal and target protein, a linkage that has previously been hypothesized as being critical^59^.

**Fig. 4:**
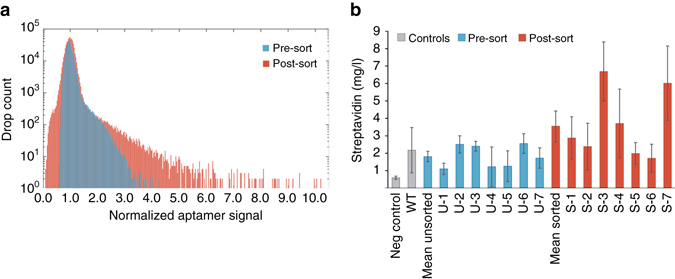
RAPID screening for the improvement of streptavidin protein secretion through an evolved secretory tag. The RAPID screening approach was used to identify mutations in the α-mating factor (αMF) secretory leader fused to streptavidin in yeast. **a** Histograms of droplet fluorescence pre-sort and post-sort (re-encapsulated) demonstrate enrichment through the process. **b** Isolated and re-transformed clones randomly selected from the pre- and post-sort populations were quantified for streptavidin production. *Error bars* represent 95% confidence intervals of biological triplicates. Protein secretion from an individual clone was increased nearly threefold over wild-type production using this approach, and there was also twofold increase in secretion between the mean of the sorted and unsorted clones

For this example, we generated a mutant library of the αMF secretion signal fused to streptavidin and transformed the resulting plasmid population into yeast. We then encapsulated and cultivated the library, using the Spinach-streptavidin aptamer for quantitation. We observed differences in streptavidin-induced fluorescence after 3 days, with a fully matured signal after five (Supplementary Fig. 10). We then sorted the library after 5 days of incubation, recovering the brightest 0.1% of droplets. To assess the screen, we re-encapsulated the sorted and unsorted libraries, and incubated and analyzed them with the same droplet fluorescence detector. The sorted pool exhibited a broad range of production phenotypes, with many variants exhibiting higher production than wild type (Fig. 4a). After re-transforming individual randomly selected variants from the sorted and unsorted populations into a fresh yeast strain, we measured streptavidin titer in supernatant samples from shake-flask production cultures and found that two of the seven variants isolated by droplet sorting have significantly increased secretion (*p* < 0.01, One-way analysis of variance (ANOVA) with Dunnett’s Test for multiple comparisons to WT control). We observed up to threefold higher supernatant streptavidin titer compared to the wild-type αMF construct and found that some mutant αMF secretory leader sequences align with previously reported mutations (Supplementary Fig. 11), with the highest performers exhibiting a nonpolar to polar mutation (I → T) in the LLFI motif^59^. In contrast, we were unable to obtain any improved mutants by randomly isolating variants from the unsorted library population. This serves as another example illustrating the efficacy and generalizability of RAPID for evolving enhanced secretion phenotypes.

## Discussion

We have demonstrated a generalizable approach for enhancing secretory phenotypes for small molecules and proteins with droplet microfluidic evolution. Our approach addresses a previously unmet need in the design-build-test cycle: the efficient testing of large secretion libraries in a format that is generalizable across diverse molecular targets. A key innovation of RAPID is implementation of Spinach RNA aptamers as general readouts for target molecule titers, which enables ultrahigh-throughput droplet assays. Indeed, aptamers are useful for a broad range of applications and are employed throughout biology. RAPID leverages this infrastructure, allowing facile construction of biosensors against new targets using published binding sequences. When no RNA aptamer sequences are available for a given target, systematic evolution of ligands by exponential enrichment (SELEX) can generate them^60^. Other extracellular biosensors, such as DNA aptamers^38^, should also be usable in our screening workflow.

A unique and valuable feature of RAPID is that it is gentle and thus capable of maintaining the secreting cells in a live and productive state for days. While this initial work focused on screening yeast libraries for enhancing extracellular metabolite or recombinant protein titers, other cell types and screening goals have been addressed using similar microfluidic sorting devices^33, 61–63^, suggesting a wide range of potential applications for our aptamer-based approach. In addition, RAPID screening yields live cells after sorting, which can be cultured or analyzed to directly recover mutational enhancements. This enables new opportunities in which in vivo mutagenesis techniques^10^ are combined with RAPID to obviate manual in vitro library generation. We envision a future in which this strategy allows design, build, and test phases to be cycled continuously without human intervention, employing automation to iterate between in vivo mutagenesis and microfluidic screening to evolve a human-defined trait of interest.

## Methods

### Microfluidic device fabrication

Soft lithography and device design (Supplementary Fig. 12) were performed using procedures described by Xia and Whitesides^64^. In brief, a layer of photoresist was spun onto silicon wafers and UV etched using a high resolution mask. The etched wafer was placed into developer solution and dried before pouring PDMS. Inlets into the embossed PDMS device were punched using a 0.75 mm biopsy tool and were attached to a glass slide using plasma bonding. Hydrophobicity was applied to the channels using aquapel (Pittsburgh Glass Works).

### Construction and testing of metabolite-binding RNA aptamers

To construct all RNA sensors, synthetic double-stranded DNA sequences (gBlocks) were obtained from IDT, incorporating the Spinach sequence with the metabolite-binding aptamer inserted into a stem loop segment^35^, as well as a T7 promoter sequence allowing transcription of the appropriate sequence (Supplementary Table 1). RNA was produced using the Ampliscribe T7-flash in vitro transcription kit using a gBlock as a template (Integrated DNA Technologies). RNA aptamers were purified by chloroform extraction using ammonium acetate.

To measure sensor fluorescence, RNA was diluted to 10 μM in 50 mM Tris-HCl, pH 7.5, 125 mM KCl, 5 mM MgCl_2_, and heat denatured at 95 °C for 3 min. It was then incubated at 37 °C for 1–2 h to allow proper folding. Finally, DFHBI or DFHBI-1T (Lucerna) was added to a final concentration of 2 μM, and the ligand was added to a final concentration of 1 mM except thrombin and streptavidin where 2.7 and 1.7 μM were used, respectively. Fluorescence was measured continuously after addition of the metabolite with an excitation wavelength of 460 nm and an emission wavelength of 500 nm for DFHBI, or with excitation of 482 nm and emission of 510 nm for DFHBI-1T.

### Testing Spinach sensors in microfluidic droplets

RNA spinach sensors, dyes, and ligands were encapsulated with droplet generation device with flow rate of 200 μl h^−1^ for both aqueous and oil phases. Fluorescence images of incubated microfluidic droplets were captured with a Zeiss Axiovert microscope (10×/0.3 Plan-NEOFLUAR objective and Zeiss FluoArc) and processed by AxioVision SE64 Rel 4.0.1.

### Growth and transformation procedures for *E. coli* and yeast

Yeast expression vectors were propagated in *E. coli* DH10β. *E. coli* strains were routinely cultivated in LB medium (10 tryptone, 5 yeast extract, and 10 g l^−1^ sodium chloride) (Teknova) at 37 °C with 225 RPM orbital shaking. LB was supplemented with 100 µg ml^−1^ ampicillin (Sigma) when needed for plasmid maintenance and propagation. Yeast strains for which maintenance of auxotrophic markers was unnecessary were propagated in YPD (10 yeast extract, 20 peptone, 20 g l^−1^ glucose). When required for plasmid maintenance, yeast strains were cultivated on a yeast synthetic complete (YSC) medium containing 6.7 g of Yeast Nitrogen Base (Difco) per l^−1^, 20 g glucose per l^−1^ and a mixture of appropriate nucleotides and amino acids (CSM, MP Biomedicals, Solon, OH). For YSC medium containing galactose, glucose was omitted from the above recipe and replaced with 20 g l^−1^ galactose. All components were supplemented with 2% agar for solid media.

For *E. coli* transformations, 25 µl of electrocompetent *E. coli* DH10β were mixed with 30 ng of ligated or Gibson-assembled DNA and electroporated (2 mm Electrporation Cuvettes (Bioexpress) with Biorad Genepulser Xcell) at 2.5 kV. Transformants were rescued for one hour at 37 °C in 1 ml SOC Buffer (Cellgro) plated on LB agar and incubated overnight. Single clones were amplified in 5 ml LB medium and incubated overnight at 37 °C. Plasmids were isolated (QIAprep Spin Miniprep Kit, Qiagen) and confirmed by Sanger sequencing.

For yeast transformations, 50 µl of chemically competent *S. cerevisiae* BY4741 (ATCC® 4002900) were transformed with 1 µg of each appropriate purified plasmid according to established protocols^65^, plated on the appropriate medium, and incubated for 3 days at 30 °C. Multiple transformations were performed as needed to generate libraries of sufficient size for directed evolution. Single colonies were picked into 1 ml of the appropriate medium and incubated at 30 °C. Plasmids were isolated from yeast using a Zymoprep Yeast Miniprep Kit II (Zymo Corporation) and transformed into *E. coli* for further amplification.

### Molecular cloning procedures

PCR reactions were performed with Q5 Hot-Start DNA Polymerase (NEB) according to manufacturer specifications. Digestions were performed according to manufacturer’s (NEB) instructions, with digestions close to the end of a linearized strand running overnight and digestions of circular strands running for 1 h at 37 °C. PCR products and digestions were purified with a QIAquick PCR Purification Kit (Qiagen). Phosphatase reactions were performed with Antarctic Phosphatase (NEB) according to manufacturer’s instructions and heat-inactivated for 15 min at 65 °C. Ligations (T4 DNA Ligase, Fermentas) were performed overnight at 22 °C followed by heat inactivation at 65 °C for 20 min. Plasmids were also made using Gibson Assembly (NEB), by mixing PCR products with the enzyme mixture and heating to 50 °C for 1 h. Ligations and Gibson-assembled plasmids were then transformed into *E. coli* DH10β and plated. Individual colonies were then amplified in liquid culture and plasmids were extracted. Correctly assembled plasmids were confirmed through restriction digestion and sequencing.

### Directed evolution for tyrosine over-production

Two libraries were made for the evolution of *aro4*: one derived from the wild-type gene and one from the K229L mutant, which has been shown to exhibit lower feedback inhibition^47^. Each gene was PCR amplified in an error-prone manner using GeneMorph II kit (Agilent) following the protocol for “medium” error-rate (4.5–9 errors per kbp). The amplified product was purified using gel extraction, then digested and cloned into a backbone plasmid as described above. The mutant plasmid libraries (ranging from ~ 5 × 10^4^ to 1 × 10^5^ in size) were then transformed into *E. coli* DH10-β using electroporation, which were then harvested from petri dishes. The plasmid libraries were then purified and transformed into a strain of yeast with *aro3* and *aro4* (both of which encode DAHP synthase activity) knocked out^43^. After transformation, the yeast libraries were grown to stationary phase and glycerol stocked.

Each library was then individually grown to saturation from frozen stock in the appropriate YSC dropout media over 2–3 days. Cells were then diluted by mixing in 100 mM Tris (pH 7.5), 200 mM KCl, 10 mM NaCl, 10 mM MgCl_2_, 30 μM RNA aptamer, 3 μM DFHBI-1T (Lucerna, Inc.), and 0.1 mg ml^−1^ of double-stranded salmon sperm DNA (Thermo Fisher) to a final cell density of 0.3. For streptavidin library we used 10 μM RNA aptamer and 1 μM DFHB1-1T. At this concentration of cells and size of droplets (40 μm), this results in ~1 in 10 droplets containing a single cell and 1 in 100 containing two cells, which may increase sorting false positives and thus affect sorting efficiency. This culture was then encapsulated in droplets and incubated at room temperature for 48 h prior to sorting.

To detect and sort microfluidic droplets using a fluorescence signal, we made use of a custom built fluorimeter and microscope. As droplets flow through the microfluidic channel, they are exposed to a 473 nm laser to excite individual droplets and the emission is measured using a PMT with a 517 nm bandpass filter. LabVIEW (National Instruments) software with a user set threshold for sorting controlled a field programmable gate array card to apply a voltage to a high voltage amplifier (Teck) to dielectrophoretically move the droplet into the sorted outlet while unsorted droplets continue unperturbed. After sorting, droplets were opened using perfluorooctanol and collected cells were cultured in dropout media for several days, at which point samples were either re-encapsulated in droplets for comparison to the pre-sort population or frozen for further assays.

Post-sort, total DNA from post-sort cultures were extracted (Zymo) and re-transformed to *E. coli*. Simultaneously, the original library was grown from glycerol stock and the same DNA extraction and transformation were carried out. Next, individual clones were picked from each, followed by plasmids isolation and sequencing. Those plasmids encoding mutations were re-transformed into a fresh strain of yeast containing the same double knockout of *aro3* and *aro4*. This strategy eliminates any possible strain adaptation that may account for high-production.

### Assaying aromatic amino acid production of cell cultures

Cells were pre-cultured in YSC dropout media for at least 2 days. The OD600 was measured, and each culture was centrifuged, washed with water, and finally diluted to OD600 = 3 with minimal media, which contains 20 glucose, 20 methionine, 12 adenine, 20 uracil, 20 histidine, and 100 mg l^−1^ leucine. Cells were incubated at 30 °C in this media for 2 additional days, and then centrifuged to pellet. The media supernatant was then measured using a tyrosine derivatization assay. 100 μl of supernatant was mixed with an equal volume of solution containing 0.05% (w/v) 1-nitroso-2-naphthol, 50% ethanol, 10% (v/v) nitric acid and 0.25 g l^−1^ (w/v) NaNO_2_. The reaction was catalyzed by incubating at 55 °C for 45 min, then read by fluorescence using an excitation of 485 nm and emission of 590 nm^45^. A standard curve of tyrosine was used to quantify. Correlation between culture supernatant tyrosine concentration (measured using this assay) and corresponding Tyr1M1 aptamer fluorescence was analyzed using GraphPad Prism 6 (Pearson r determination, *α* = 0.05 for significance).

### Characterization of exponential growth rates

Maximum rates of exponential growth were characterized for cells expressing mutant *aro4* genes using a Bioscreen C (Growth Curves USA). Briefly, selected strains were inoculated into the appropriate media, either complete (YSC-Histidine) or minimal (20 glucose, 20 methionine, 12 adenine, 20 uracil, and 100 mg l^−1^ leucine), at OD600 = 0.1 and OD600 measurements were taken every 15 min using continuous shaking for 3 days at 30 °C. Growth rates were calculated using a custom MATLAB script (available upon request).

### Directed evolution for streptavidin secretion

The alpha mating factor (αMF) secretion signal from *S. cerevisiae* was fused to full-length streptavidin (SA, including PDB 2BC3_A residues 12-159) and codon optimized for *S. cerevisiae* expression as a gBlock (Integrated DNA Technologies). SA was PCR amplified and cloned into p426-GAL1^66^ to yield p426-GAL1-SA. The αMF sequence was then mutated (full length except ATG start codon) using nucleotide analog mutagenesis (NAM) according to the general dNTP/8-oxo-dGTP (Trilink) protocol described by Zaccolo et al.^67^. Specifically, 20 μM of nucleotide analogs was used in a reaction with Taq polymerase (NEB) with a varying number of PCR cycles (12–30 cycles). The mutated PCR product was then amplified using Q5 High Fidelity Polymerase (NEB) and the same primers in order to clear the nucleotide analogs and amplify sufficient DNA for Gibson assembly^4^. The resulting PCR product was then combined with PCR linearized acceptor vector p426-GAL1-SA in 10 standard Gibson assembly reactions to yield the plasmid library/pool p426-GAL1-αMF[NAM]-SA. This library was then transformed into electrocompetent DH10β in 25 transformations to yield a library size of ~5 × 10^6^ variants. After MaxiPrep (Zymo) to recover the plasmid library from *E. coli*, the library was transformed into *S. cerevisiae* BY4741 using the standard lithium acetate transformation protocol. This yielded an estimated yeast library size of 1–3 × 10^6^ variants based on fractional plating and colony counting on YSC-Ura + Glucose selective plates. A sample of the unsorted/pre-sort library was subjected to yeast MiniPrep (Zymo), and the unsorted pool was transformed into electrocompetent DH10β for recovery and Sanger sequencing of seven clonal colonies. This same procedure (yeast MiniPrep, DH10β transformation, *E. coli* MiniPrep, Sanger sequencing of) was also used to later isolate individual sequences from seven clonal colonies derived from the RAPID sorted pool. RAPID sorting was performed as described above for tyrosine directed evolution. After sorting, droplets were opened using perfluorooctanol and collected cells were cultured in dropout media for several days, at which point samples were either re-encapsulated in droplets for comparison to the pre-sort population or frozen for further assays.

High-copy episomal 2μ plasmids (p426-GAL1 based) bearing individual mutant αMF sequences or sequence pools (unsorted and sorted) were re-transformed into fresh *S. cerevisiae* BY4741 cells using the Frozen EZ II Transformation Kit (Zymo) according to the manufacturer’s protocol scaled down to 96-well plate format: 20 μl cells (pre-frozen), 2 μl plasmid DNA, and 200 μl EZ III Buffer per well. The wild-type αMF-SA plasmid (WT, no mutagenesis), a negative control plasmid (p426-GAL1 with no streptavidin gene), the unsorted plasmid pool, and the sorted plasmid pool were also re-transformed at the same time to act as controls/comparators for the individual clone sequences. After plating each transformation mixture on YSC-Ura + Glucose for selection, three colonies were picked from each plate (biological triplicate) into 1 ml of liquid YSC-Ura media containing glucose for cell outgrowth and glycerol stocking in 96-deepwell blocks. Starter cultures were then inoculated from glycerol stocks into 2 ml of YSC-Ura + Glucose for 3 days of growth at 30 °C in 14-ml culture tubes in a rotating roller drum. All 2 ml of culture was then added to 58 ml of YSC-Ura + 2%Glucose, and the 60 ml cultures were incubated in 250-mL shake flasks at 225 RPM/30 °C for 4 days (final OD600 = 5–7). Sufficient cells to seed 30 ml cultures at OD600 = 10 were spun down at 500 x g for 10 min, then re-suspended in 25 ml of YSC-Ura + 4%Galactose to induce protein production at 225 r.p.m./30 °C in a shaking incubator for 5 days. 20 ml of supernatant was then harvested by gentle centrifugation (500×*g* for 10 min) and an equal volume of saturated ammonium sulfate solution (~ 4.32 M, sterile filtered to remove excess solid) was added to precipitate the secreted streptavidin overnight at 2–8 °C. After centrifugation at 15,000×*g* for 1 h at 4 °C, the resulting precipitated protein pellet was re-suspended until visibly homogeneous in 4 ml of Corning DPBS (i.e., 5× concentration relative to production culture volume) and allowed to rehydrate overnight at 2–8 °C in DPBS buffer before assay. Reported concentrations are corrected for 5× concentration factor in purification and represent titer in the culture supernatant.

The Spinach2-Streptavidin aptamer was ordered as a synthetic DNA gBlock (Integrated DNA Technologies). Aptamer RNA was then prepared in a similar manner to the previously described tyrosine aptamer. Briefly: the Spinach2-Streptavidin biosensor sequence was PCR amplified with a primer set to add an upstream T7 promoter for in vitro transcription. The resulting PCR product was purified by spin column (Thermo Scientific) and used as a template for in vitro T7 transcription using the AmpliScribe T7-Flash Transcription kit (Epicentre) according to the manufacturer protocol. Pure RNA was isolated by standard ethanol precipitation. 1, 5, 10, 20, 30, 40, 50, 60 and 100 mg l^−1^ of streptavidin standard (NEB) were chosen as standard curve points (Supplementary Fig. 13). To make a standard curve sample, streptavidin stock (100 mg l^−1^) was diluted directly in 1× DPBS (Corning) to the target concentration. Secreted streptavidin samples from cultures were ammonium sulfate precipitated and re-suspended in DPBS before assay. Each sample (standard curve point or re-suspended sample) was mixed with a solution of 10 μM Spinach2-Streptavidin aptamer and 2 μM DFHBI-1T (Lucerna, Inc.) and incubated at 25 °C for 14 h in a buffer containing 100 mM Tris, pH 7.5, 200 mM KCl, 10 mM NaCl and 1 mM MgCl_2_. Total volume of reaction was 100 μl and the fluorescence assay was carried out in a black wall 96-well microtiter plate (Thermo). Fluorescence measurements were performed using a Cytation3 imaging reader (BioTek) using the following instrument parameters: excitation wavelength = 482 nm, emission wavelength = 510 nm, gain = 100, read height = 7 mm. The secreted streptavidin concentration in yeast production cultures was determined based on a linear regression of the standard curve from 1–40 mg l^−1^ (all tested samples fell in this range of fluorescence signal) and by correcting for a 5× concentration factor from protein precipitation. Statistical analysis of mutants was performed using GraphPad Prism 6 (one-way ANOVA with Dunnett’s Test for multiple comparisons to the WT control). A test for equal variances was performed (GraphPad Prism 6) to confirm that variances were not significantly different prior to proceeding with One-way ANOVA and Dunnett’s Test.

### Data availability

Data that support the findings of this study are available from the corresponding authors upon request.

## Electronic supplementary material

Supplementary Information
