# Supplementary material for: RNA-aptamers-in-droplets (RAPID) high-throughput screening for secretory phenotypes
Source: Nat Commun. 2017 Aug 23;8:332. doi: 10.1038/s41467-017-00425-7 (PMC5569033; doi:10.1038/s41467-017-00425-7)
Supplement: Supplementary file 1 — Supplementary Information [file 41467_2017_425_MOESM1_ESM.pdf]

File name: Supplementary Information
Description: Supplementary Figures, Supplementary Tables and Supplementary References.

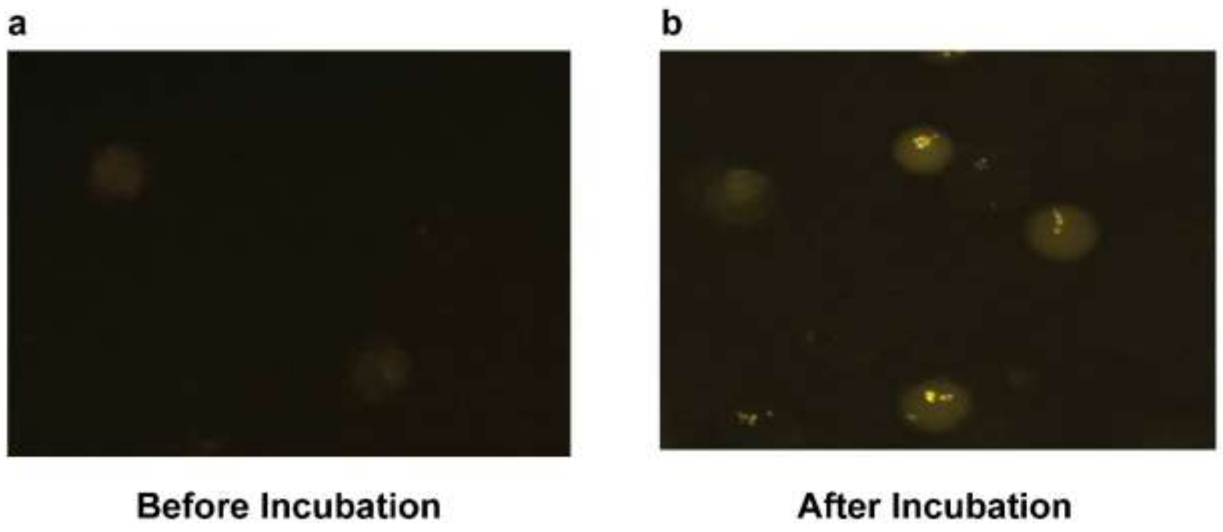

Supplementary Figure 1. *40 micron droplets were generated containing on average a single yeast cell (Poisson distribution) secreting yECitrine*. (a) Before incubation, droplets are dim and required increased gain to properly visualize droplets using fluorescence microscopy. (b) After incubation, individual yeast cells in droplets have multiplied and a fluorescence signal was detected from both the cell and the surrounding media as yECitrine was produced and secreted.

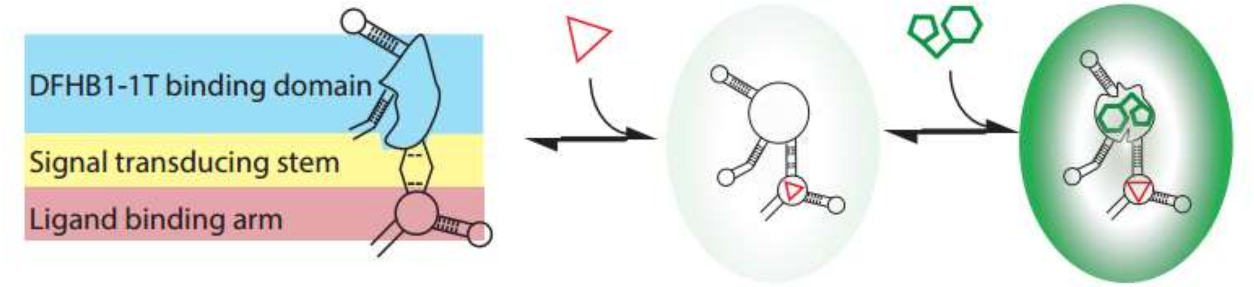

Supplementary Figure 2. *Structural organization of Spinach Aptamers*. RNA and DNA aptamers can be designed to bind specific proteins or small molecules with high specificity and selectivity. Spinach aptamers make use of an exogenous dye to fluoresce brightly upon binding, partly by making use of a unique G-quadruplex motif that serves as a platform for fluorophore binding[1]. The thermodynamic stability of the G-quadruplex structure is higher in RNA[2], as the RNA structure is less hydrated than the DNA structure. In addition, RNA has an additional 2' hydoxyl group in the ribose sugar allowing for enhanced stability of the RNA G-quadruplex structure[3]. Modified Spinach aptamers combine a ligand binding arm with a signal transducing stem to fluoresce brightly upon binding of both ligand and dye. Note that structural re-arrangements between the ligand binding arm and DFHBI-1T binding domain in the signal transducing stem are necessary to tightly bind dye and develop a signal after ligand binding. Additional information on Spinach aptamer modifications has been previously published by Strack *et al*[4].

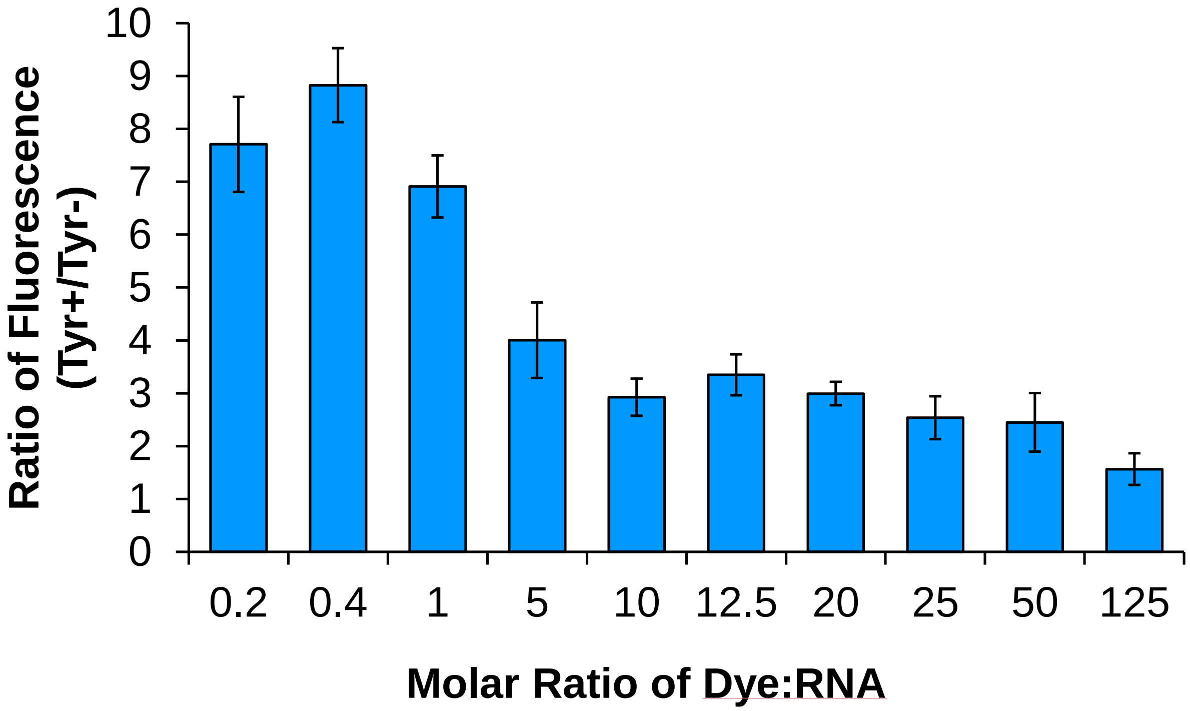

Supplementary Figure 3. *The ratio of fluorescence signal in the presence of tyrosine to fluorescence signal in the absence of tyrosine (Tyr+/Tyr-) can be tuned by varying the dye to RNA aptamer ratio (Dye:RNA).* Values represent the mean of each condition prepared in triplicate, and error bars depict standard deviation.

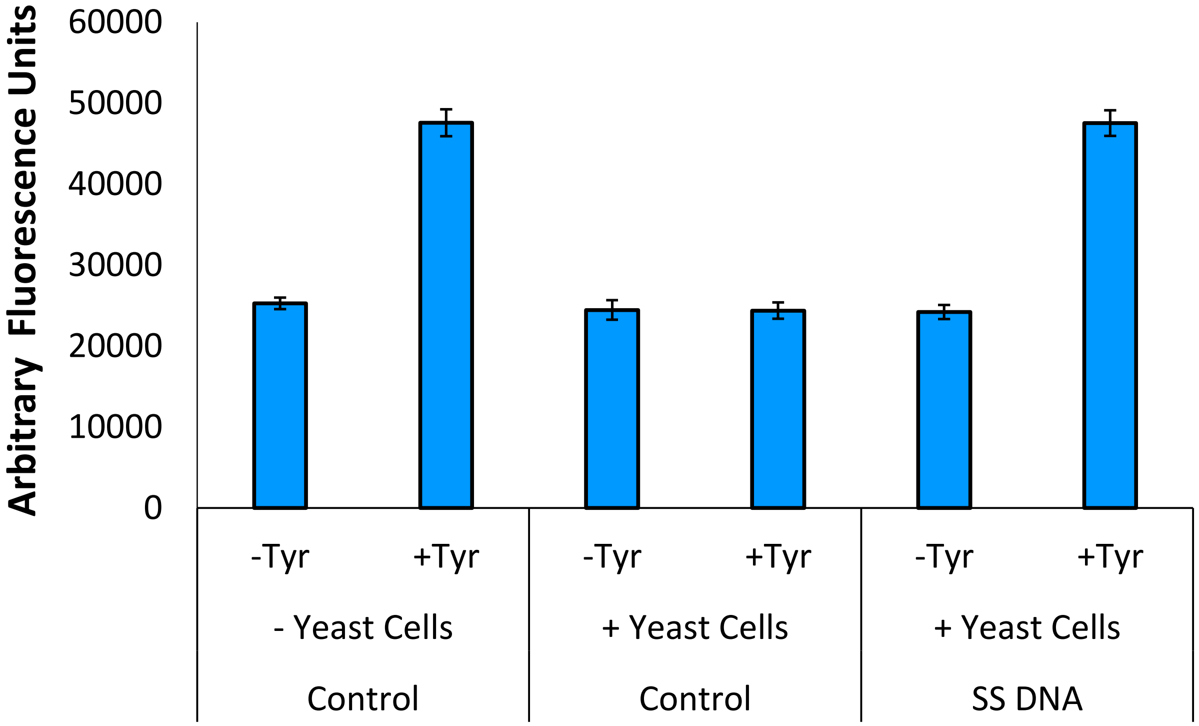

Supplementary Figure 4. *Salmon sperm DNA can negate the loss of aptamer signal from incubation with yeast cells*. Incubating aptamers with yeast cells resulted in a dimming of fluorescence to a level below that of even negative control samples. We postulated that this effect may be due to cellular lipids binding to charged substances such as the RNA aptamer. By washing and incubating the yeast cells with salmon sperm DNA (SS DNA), we were able to recover aptamer signal from 1 mM Tyrosine. Values represent the mean of each condition prepared in triplicate, and error bars depict standard deviation.

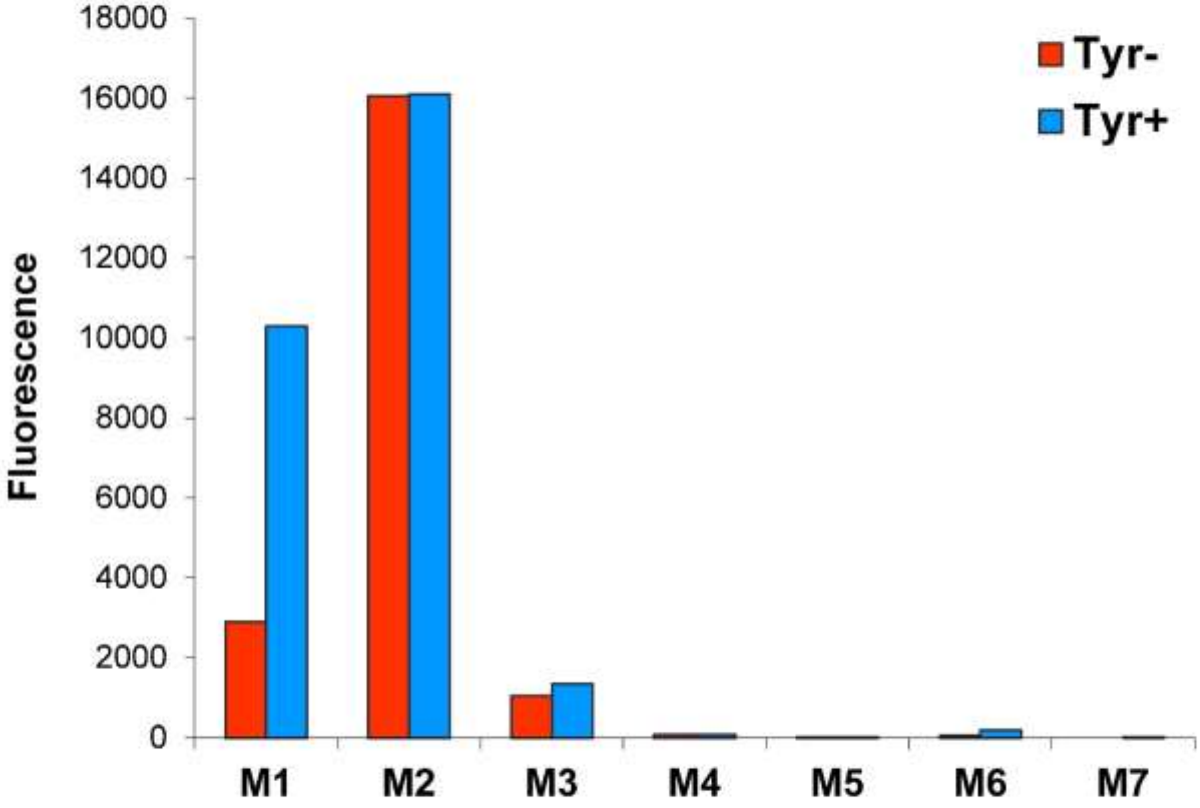

Supplementary Figure 5. *Initial testing of stem variants for aptamer-based tyrosine detection*. Tyrosine-sensing aptamer configurations were preliminarily screened (N = 1) in the presence of tyrosine (Tyr+) and in the absence of tyrosine (Tyr-). While not the brightest signal, the Tyr1 M1 aptamer yielded a sufficiently high fluorescence signal and the desired signal induction by tyrosine. Based on this preliminary screen, Tyr1 M1 was selected for further characterization and application.

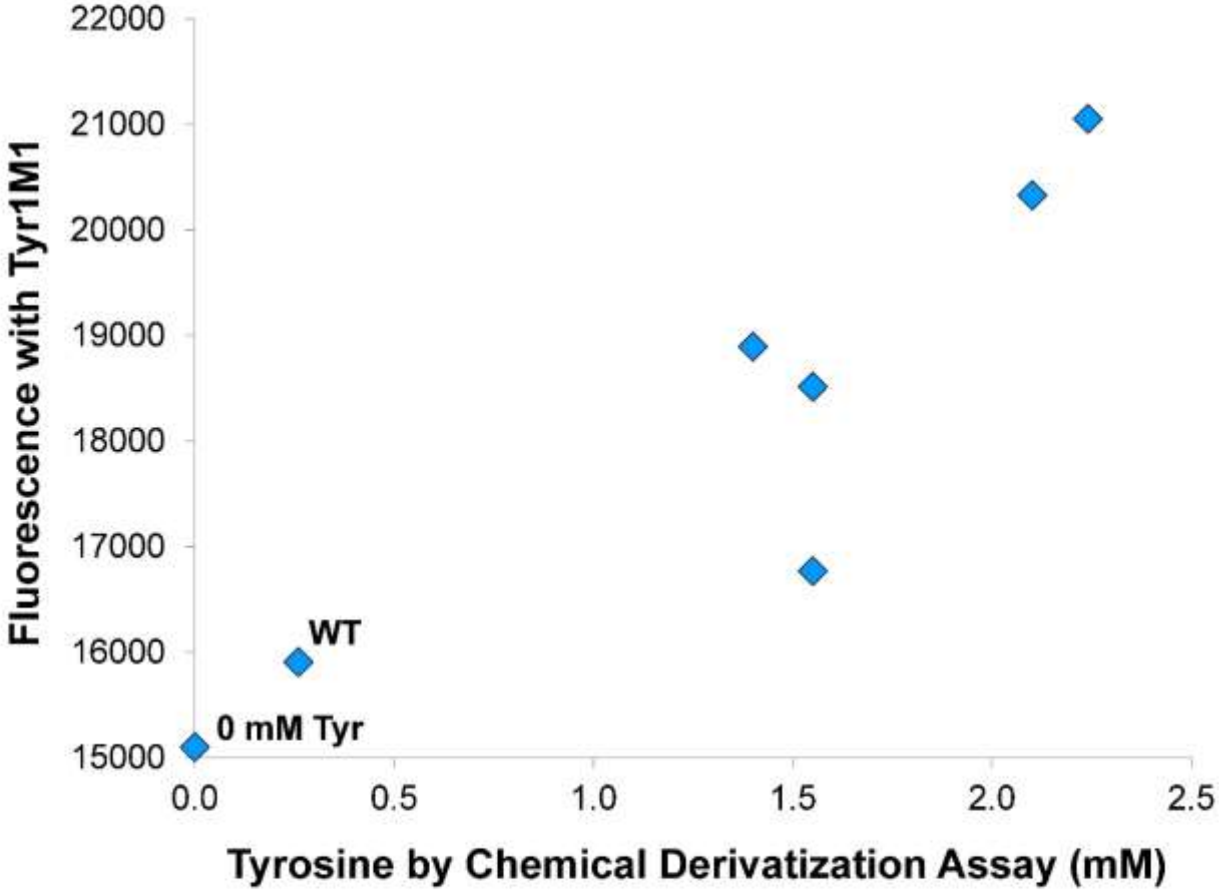

Supplementary Figure 6. *Tyrosine-sensing aptamer fluorescence is correlated with extracellular tyrosine concentration*. Supernatants from yeast cultures were assayed for tyrosine concentration by chemical derivatization and for aptamer-induced fluorescence. Correlation (Pearson r = 0.9175, p = 0.0036, N = 1 for 7 XY pairs) was observed between tyrosine concentration and Tyr1M1 fluorescence for samples from a panel of previously engineered tyrosine producing yeast strains[5].

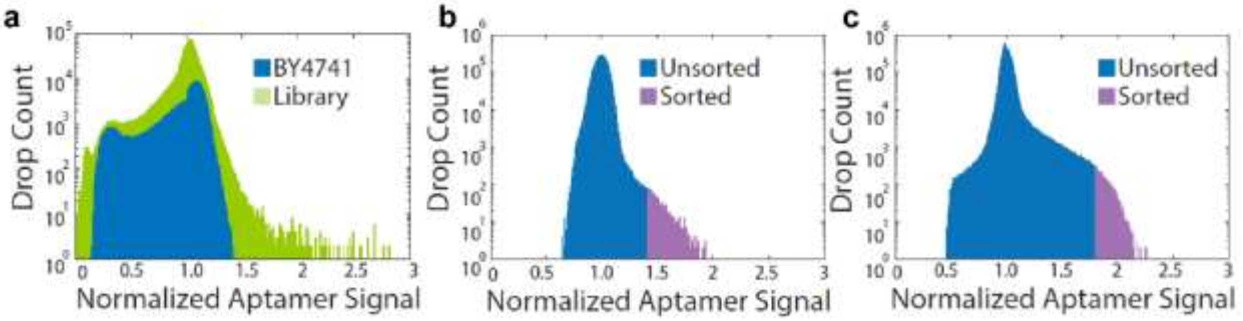

Supplementary Figure 7. *Sorting parameters for aro4 and aro4-229 libraries*. (a) Droplets were incubated offline for three days for a signal to be detected between droplets containing BY4741 and those containing an *aro4* library. (b) A total of 3,817,741 drops were screened for the *aro4* library on the third day and the top 1,000 droplets were sorted. (c) A total of 5,296,644 drops were screened for the *aro4-K229L* library on the third day and the top 3,000 droplets were sorted.

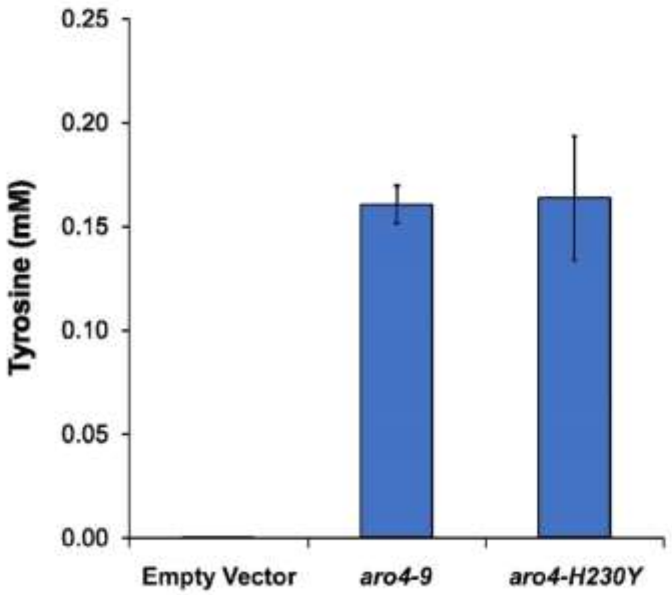

Supplementary Figure 8. *Tyrosine production from yeast transformed with an empty vector, mutant* aro4-9*, and single point mutant* aro4-H230Y. The single point mutation at H230Y in *aro4* can fully recapitulate the tyrosine production phenotype observed with *aro4-9*. Values represent the mean of 3 biological replicates from plasmid transformation, and error bars depict the standard error of the mean (±SEM).

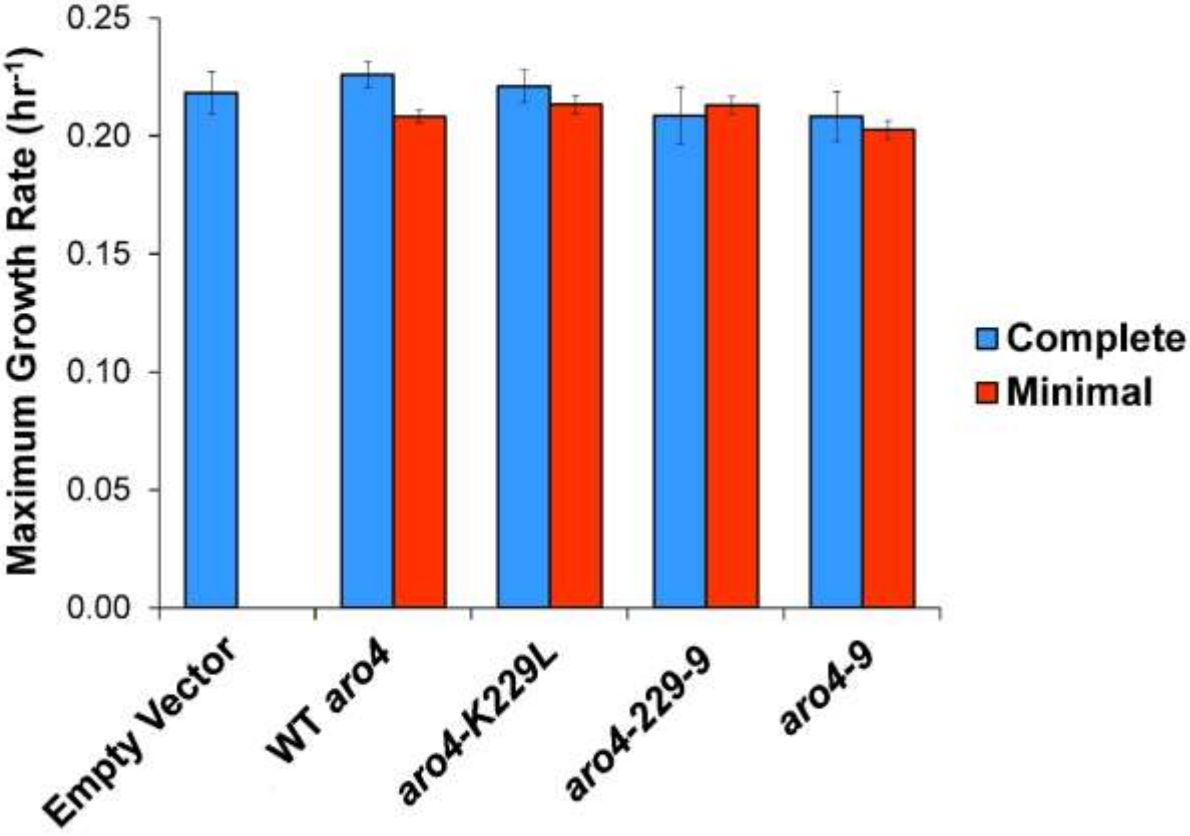

Supplementary Figure 9. *Maximum exponential growth rate of yeast strains transformed with an empty vector, WT* aro4*,* aro4-K229L*,* aro4-229-9*, and* aro4-9 *in minimal or complete medium*. There is no significant difference in the growth rates of these strains in either medium formulation. Values represent the mean of 3 biological replicates from plasmid transformation, and error bars depict the standard error of the mean (±SEM).

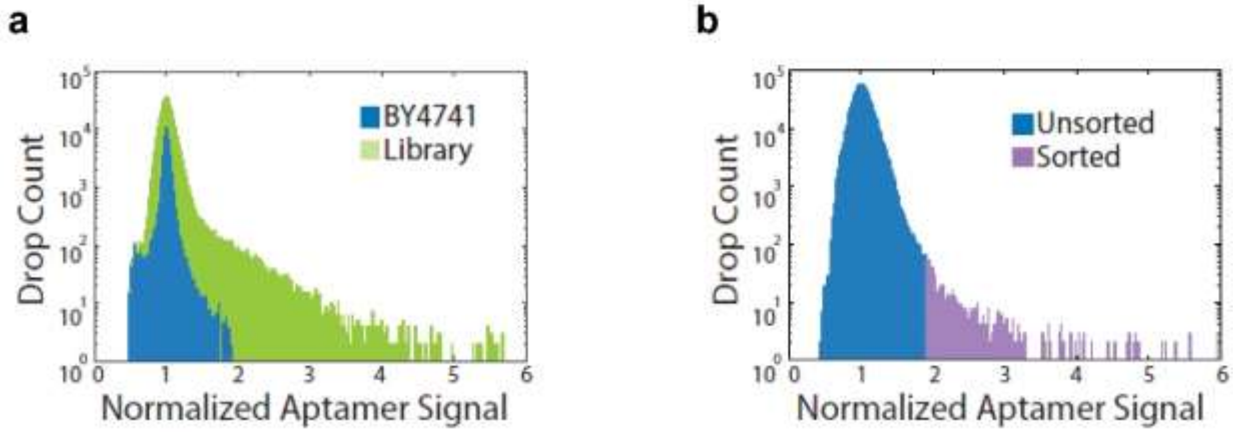

Supplementary Figure 10. *Parameters for screening and sorting of streptavidin with a library of secretion tags*. (a) Droplets were incubated offline for five days to develop the fluorescence signal to be detected between droplets containing BY4741 and those containing streptavidin fused to the αMF secretion signal library. (b) A total of 995,663 droplets were screened on the fifth day and the top 1000 droplets were sorted.

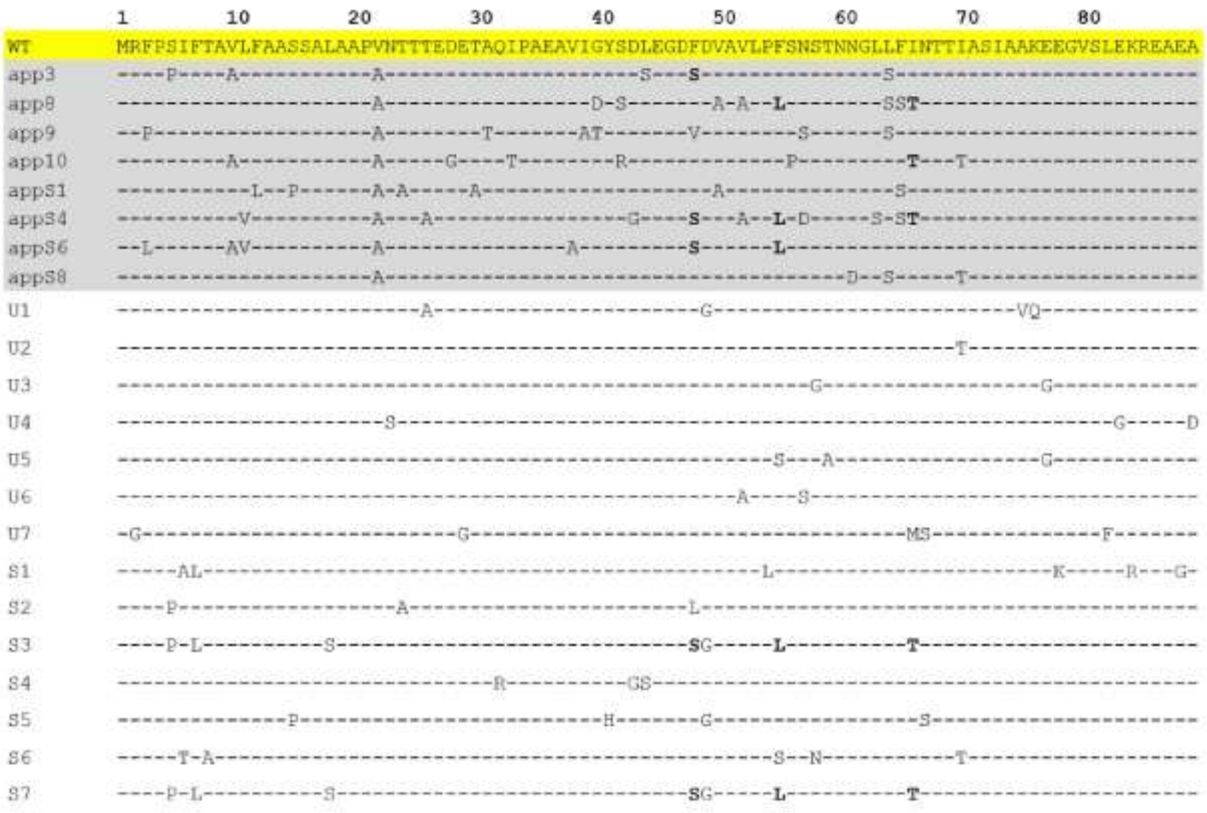

Supplementary Figure 11. *Comparison of isolated secretion leader sequences*. WT αMF secretory leader sequence (top line in yellow) compared to previously isolated mutant sequences (in gray, denoted appXX)[6] and mutants isolated in this work from the un-sorted library population (UX) or from the RAPID sorted population (SX).

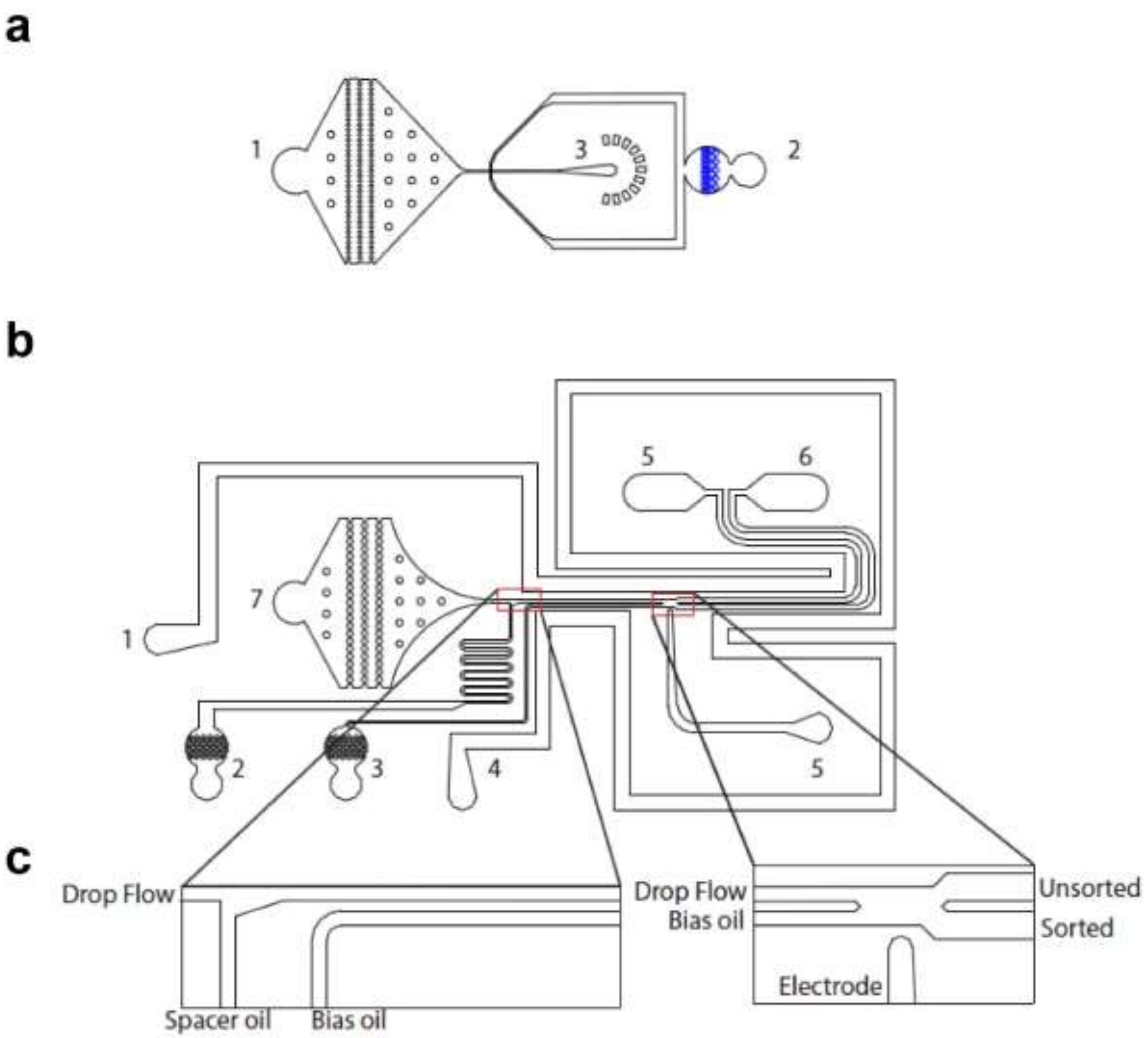

Supplementary Figure 12. *Microfluidic devices utilized for RAPID.* (a) A flow focused drop generator is used to make 40 micron droplets. Labelled components: (1) inlet (2) oil inlet (3) outlet. (b) A microfluidic sorter schematic is depicted, where drops are re-injected and their flow through the channel is affected through a bias and spacer oil flow. An electrode applies a square wave of pulses through a LabVIEW command software (National Instruments) to alter the direction of high fluorescence droplets toward the sorted outlet. The labelled parts of the device are (1) moat solution inlet (2) spacer oil (3) bias oil (4) moat solution inlet (5) unsorted droplet outlet (6) sorted droplet outlet (7) re-injection port. (c) Details of the droplet flow towards the sorting electrode are depicted, with specific elements labeled.

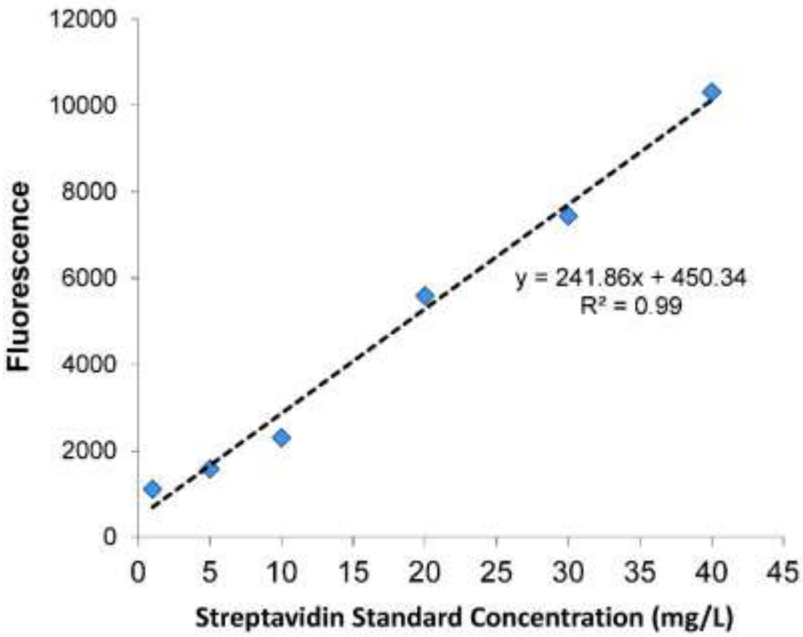

Supplementary Figure 13. *Purified streptavidin standard curve for microtiter plate assay of streptavidin concentration using the Spinach-streptavidin aptamer*. This standard curve was generated within the same assay plate (N=1 for six standard solution concentrations) as all streptavidin-containing samples depicted in Figure 4b and was used to calculate culture supernatant concentrations.

Aptamer Name	Target Molecule	RNA Sensor Template Sequence
Tyr1M1	Tyrosine / Tryptophan / Phenylalanine	**GAAATTAATACGACTCACTATA***GGATGTAACTGAATGAAATGGTGAAGGACGGGTCCA***GCCCTTGGCAGTCAACTCGTGCGATCGTGAAAACGGGGCAAGATGGCCTTACAGCGGTCAATACGGGGGTCATCAGATAGGGAGGCCAGGATA***TGTTGAGTAGAGTGTGAGCTCCGTAACTAGTTACATC*
Tyr1M2	Tyrosine / Tryptophan / Phenylalanine	**GAAATTAATACGACTCACTATA***GGATGTAACTGAATGAAATGGTGAAGGACGGGTCCA***AACGTGAAGGCAGTCAACTCGTGCGATCGTGAAAACGGGGCAAGATGGCCTTACAGCGGTCAATACGGGGGTCATCAGATAGGGAGGCCTTGGCGT***TGTTGAGTAGAGTGTGAGCTCCGTAACTAGTTACATC*
Tyr1M3	Tyrosine / Tryptophan / Phenylalanine	**GAAATTAATACGACTCACTATA***GGATGTAACTGAATGAAATGGTGAAGGACGGGTCCA***CAGCATAGGCAGTCAACTCGTGCGATCGTGAAAACGGGGCAAGATGGCCTTACAGCGGTCAATACGGGGGTCATCAGATAGGGAGGCCTAGAGCAACA***TGTTGAGTAGAGTGTGAGCTCCGTAACTAGTTACATC*
Tyr1M4	Tyrosine / Tryptophan / Phenylalanine	**GAAATTAATACGACTCACTATA***GGATGTAACTGAATGAAATGGTGAAGGACGGGTCCA***AGACATTTTGGCGGCAGTCAACTCGTGCGATCGTGAAAACGGGGCAAGATGGCCTTACAGCGGTCAATACGGGGGTCATCAGATAGGGAGGCCGACATGA***TGTTGAGTAGAGTGTGAGCTCCGTAACTAGTTACATC*
Tyr1M5	Tyrosine / Tryptophan / Phenylalanine	**GAAATTAATACGACTCACTATA***GGATGTAACTGAATGAAATGGTGAAGGACGGGTCCA****TATGCACTCGTTGTGGCAGTCAACTCGTGCGATCGTGAAAACGGGGCAAGATGGCCTTACAGCGGTCAATACGGGGGTCATCAGATAGGGAGGCCACTAGT****TGTTGAGTAGAGTGTGAGCTCCGTAACTAGTTACATC*
Tyr1M6	Tyrosine / Tryptophan / Phenylalanine	**GAAATTAATACGACTCACTATA***GGATGTAACTGAATGAAATGGTGAAGGACGGGTCCA***GCGTTAAAGGGCAGTCAACTCGTGCGATCGTGAAAACGGGGCAAGATGGCCTTACAGCGGTCAATACGGGGGTCATCAGATAGGGAGGCCGATGGA***TGTTGAGTAGAGTGTGAGCTCCGTAACTAGTTACATC*
Tyr1M7	Tyrosine / Tryptophan / Phenylalanine	**GAAATTAATACGACTCACTATA***GGATGTAACTGAATGAAATGGTGAAGGACGGGTCCA***CTCCTCGAAAGGCAGTCAACTCGTGCGATCGTGAAAACGGGGCAAGATGGCCTTACAGCGGTCAATACGGGGGTCATCAGATAGGGAGGCC***TGTTGAGTAGAGTGTGAGCTCCGTAACTAGTTACATC*
SAM	S-Adenosyl Methionine	**GAAATTAATACGACTCACTATA***GGACGCGACTGAATGAAATGGTGAAGGACGGGTCCA***CGAAAGGATGGCGGAAACGCCAGATGCCTTGTAACCGAAAGGGT***TGTTGAGTAGAGTGTGAGCTCCGTAACTAGTCGCGTC*
Thrombin	Thrombin	**GAAATTAATACGACTCACTATA***GATGTAACTGAATGAAATGGTGAAGGACGGGTCCA***GGAACAAAGCTGAAGTACTTACCT***TGTTGAGTAGAGTGTGAGCTCCGTAACTAGTTACATC*
Streptavidin (SA)	Streptavidin	**GAAATTAATACGACTCACTATA***GATGTAACTGAATGAAATGGTGAAGGACGGGTCCA***CGACCGACCAGAATCATGCAAGTGCGTAAGATAGTCGCGGGCCGGGGT***TGTTGAGTAGAGTGTGAGCTCCGTAACTAGTTACATC*

Supplementary Table 1. DNA templates used for T7 *in vitro* transcription of RNA aptamers. **Bold** bases indicate the T7 promoter, *italicized* bases indicate the Spinach/Spinach2 scaffold domains, and **bold underlined** bases indicate the variable target binding domain.

**Mutagenesis Library**	**Pre-sort % positive**	**Post-sort % positive**	**Pre-sort mean**	**Post-sort mean**
Aro4p (regulatory region)	0.02	0.06	1.5	1.9
Aro4p K229L (whole gene)	0.02	0.12	2.0	2.4
αMF-Streptavidin (secretory leader)	0.02	0.21	3.9	4.5

Supplementary Table 2. Percent positive and mean fluorescence for gated sub-population of highly fluorescent droplets. The gate defining the sub-population was set to contain the top 0.02% of each 'pre-sort' droplet population and was then applied to the 'post-sort' droplets.

Supplementary References

1 Warner, K. D. *et al.* Structural basis for activity of highly efficient RNA mimics of green fluorescent protein. *Nature structural & molecular biology* **21**, 658-663, doi:10.1038/nsmb.2865 (2014).

2 Bhattacharyya, D., Mirihana Arachchilage, G. & Basu, S. Metal Cations in G-Quadruplex Folding and Stability. *Frontiers in Chemistry* **4**, doi:10.3389/fchem.2016.00038 (2016).

3 Sacca, B., Lacroix, L. & Mergny, J. L. The effect of chemical modifications on the thermal stability of different G-quadruplex-forming oligonucleotides. *Nucleic Acids Res* **33**, 1182-1192, doi:10.1093/nar/gki257 (2005).

4 Strack, R. L., Song, W. & Jaffrey, S. R. Using Spinach-based sensors for fluorescence imaging of intracellular metabolites and proteins in living bacteria. *Nat Protoc* **9**, 146-155, doi:10.1038/nprot.2014.001 (2014).

5 Leavitt, J. M. *et al.* Biosensor-Enabled Directed Evolution to Improve Muconic Acid Production in Saccharomyces cerevisiae. *Biotechnology journal*, doi:10.1002/biot.201600687 (2017).

6 Rakestraw, J. A., Sazinsky, S. L., Piatesi, A., Antipov, E. & Wittrup, K. D. Directed evolution of a secretory leader for the improved expression of heterologous proteins and full-length antibodies in Saccharomyces cerevisiae. *Biotechnol Bioeng* **103**, 1192-1201, doi:10.1002/bit.22338 (2009).
